# Diabetes alters vascular mechanotransduction data: Pressure-induced regulation of Nf-kapa-B p65 and translational associated signaling in the rat inferior vena cava

**DOI:** 10.1016/j.dib.2017.08.025

**Published:** 2017-09-01

**Authors:** Kevin M. Rice, Nandini D.P.K. Manne, Ravikumar Arvapalli, Gautam K. Ginjupalli, Eric R. Blough

**Affiliations:** a Center for Diagnostic Nanosystems, Marshall University, Huntington, WV, USA; b Department of Internal Medicine, Joan C. Edwards School of Medicine, Marshall University, Huntington, WV, USA; c Biotechnology Graduate Program West Virginia State University, Institute, WV, USA; d Department of Health and Human Service, School of Kinesiology, Marshall University, Huntington, WV, USA; e Department of Public Heath, Marshall University, Huntington, WV, USA; f Department of Pharmaceutical Sciences and Research, School of Pharmacy, Marshall University, Huntington, WV, USA; g Department of Pharmacology, Physiology and Toxicology, Joan C. Edwards School of Medicine, Marshall University, Huntington, WV, USA

## Abstract

Diabetic patients have a high rate of vein graft failure due to attrition or vessel occlusion that cause recurrent ischemic events or vein graft. Veins grafted into a high-pressure arterial environment must undergo vascular remodeling to better handle the altered hemodynamics and intravascular increased pressure. Multiple cellular and molecular events are purported to be associated with vascular remodeling of veins. Understanding the effect diabetes has on vascular mechano-transductive response is critical to decreasing graft failure rates. This article represents data regarding a study published in Cardiovascular Diabetology [1] and Open Journal of Endocrine and Metabolic Diseases [2] with the purpose of evaluating the effect of pressurization on rat inferior venae cavae (IVC). Here we provide the information about the method and processing of raw data related to our prior publish work and Data in Brief articles [3], [4]. The data contained in this article evaluates the contribution of NF-kB signaling and associated proteins. IVC from lean and obese animals were exposed to a 30 min of perfusion at 120 mm Hg pressure and evaluated for changes in expression and (IkB-alpha, NF-kB p50, NF-kB p105, NF-kB p65, Traf2, caspase 12), phosphorylation of (IkB-alpha (ser 32), Fox01 (ser 256), and Fox04 (ser 193)) proteins thought to be involved in the regulation of vascular mechanotransduction.

**Specifications Table**

**Table:** 

Subject area	*Biology*
More specific subject area	*Cardiovascular diabetic surgical tissue response*
Type of data	*graph, figure*
How data was acquired	*immunoblotting*
Data format	*analyzed*
Experimental factors	*IVC mounted vessels were subjected to 120* *mm Hg of pressure for 30 minutes. Protein was then isolated from tissue for western blot analysis.*
Experimental features	*IVC obtained from Lean and Obese male Zucker rats were used in this experiment*
Data source location	*Huntington, WV USA*
Data accessibility	*Data is with this article and is related to articles published and in review*[1], [2], [3], [4]

**Value of the data**

• The data presented in this Brief is vital to understanding the effect of diabetes on venous mechanotransduction.
• This data gives insight into the how diabetes alters tissue response to stimuli.
• This data provides a more thorough understanding of the NF-kB involvement in pressure mediated signaling in both diabetic and non-diabetic venous tissue.

## 1 Data

### 1.1 NF-kB p50 and p105

**Fig. 1:**
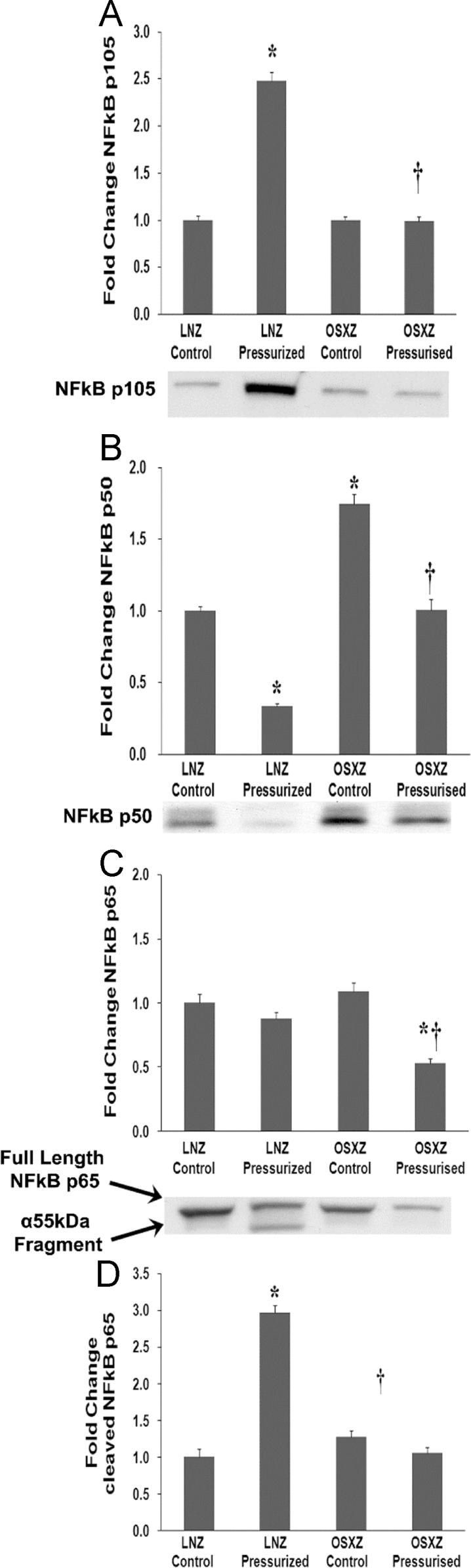
Diabetes alters loading-induced NFkB p105, NFkB p50, and NFkB p65 expression rat inferior vena cava. The basal (control) and pressure-induced expression of NFkB p105, NFkB p50, and NFkB p65 in venae cavae from non-diabetic lean Zucker (LNZ) and diabetic obese syndrome X Zucker (OSXZ) rats. * Significantly different from unloaded venae cavae within the same group (p< 0.05). † Significantly different from corresponding LNZ venae cavae (p< 0.05). n = 6/group.

To determine the effect of pressurization of inferior vena cava (IVC) from diabetic male obese syndrome-X Zucker (OSXZ) diabetic and nondiabetic male normal lean Zucker (LNZ) animals we evaluated the expression of nuclear factor kappa-light-chain-enhancer of activated B cells (NF-kB p50 (NFkB2) [5], [6]. IVCs obtained from the OSXZ control group showed no significant difference in the expression of NF-kB p105 when compared to the LNZ control animals. Pressurization resulted in a significant increase in NF-kB p105 in the LNZ IVC (148 ± 9.3%, p<0.05) but did not illicit an increase in the levels of NF-kB p105 in the OSXZ IVC (Fig. 1-A). Compared to LNZ controls, NF-kB p50 was elevated in the OSXZ control IVC (74 ± 7.1%, p<0.05). Pressurization of the IVC resulted in a significant decrease in the expression of NF-kB p50 in the LNZ IVC (66 ± 1.8% p<0.05) and the OSXZ IVC (74 ± 7.5%, p<0.05). The level of NF-kB p50 after pressurization in the OSXZ was not significantly different from the basal level of the LNZ control vessels (Fig. 1-B).

### 1.2 NF-kB p65 cleavage

Examination of the inferior vena cava expression of NF-kB p65 demonstrated no significant difference between the LNZ and OSXZ control animals. Pressurization had no effect on the level of NF-kB p65 in the LNZ IVC but produced a significant decrease in the OSXZ IVC (55 ± 3.5%, p<0.05) (Fig. 1-C). The cleavage of NF-kB p65 [7], [8], [9], [10] is thought to potentiate apoptosis [11]. NF-kB p65 cleavage was increased by (197 ± 21.3%, p<0.05) in the pressurized IVC obtained from LNZ animals when compared to control OSXZ animals. No significant difference was detected in the basal expression of cleaved NF-kB p65 between the IVC obtained from LNZ and OSXZ animals (Fig. 1-D).

### 1.3 IkB-alpha

**Fig. 2:**
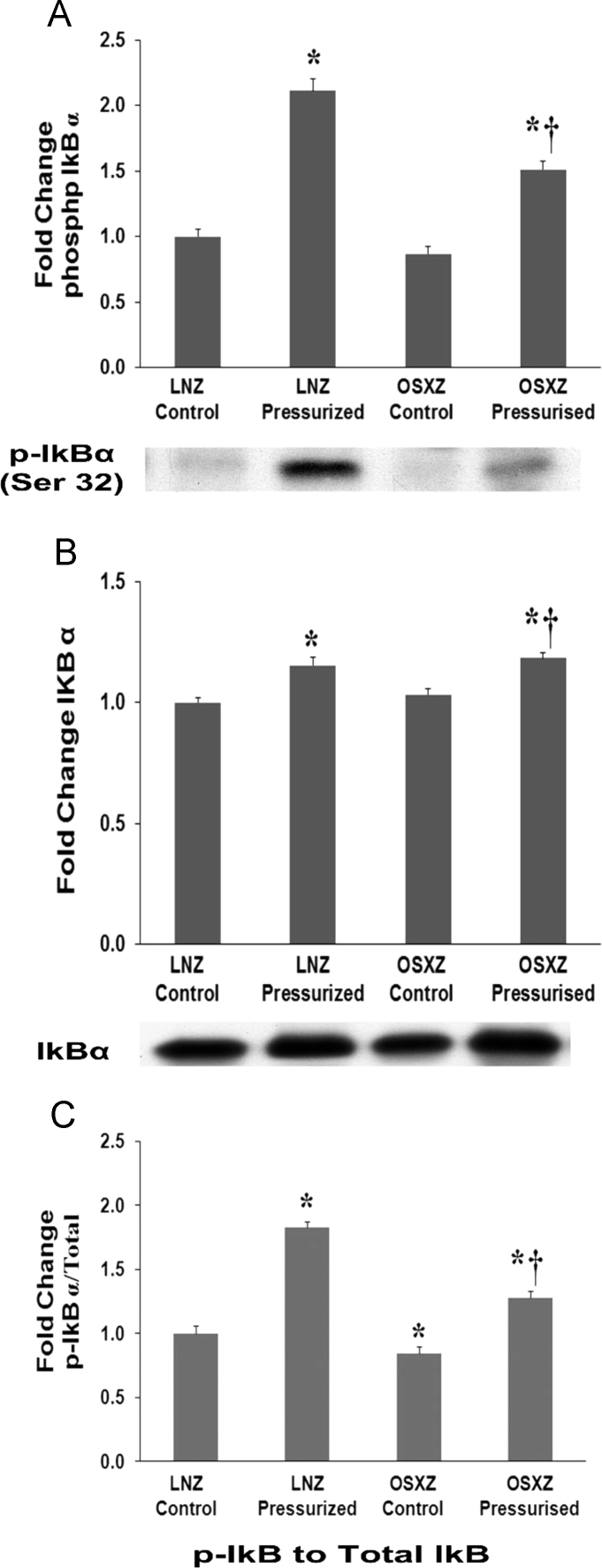
Diabetes alters loading-induced IkB-alpha expression and phosphorylation in rat inferior vena cava. The basal (control) and pressure-induced expression and phosphorylation of IkB-alpha in venae cavae from non-diabetic lean Zucker (LNZ) and diabetic obese syndrome X Zucker (OSXZ) rats. * Significantly different from unloaded venae cavae within the same group (p< 0.05). † Significantly different from corresponding LNZ venae cavae (p< 0.05). n = 6/group.

The basal nuclear factor of kappa light polypeptide gene enhancer in B-cells inhibitor, alpha (IkB-α) [12], [13] levels observed in OSXZ IVC were not significantly different when compared to the LNZ control. Pressurization resulted in a slight but significant increase in IkB expression in both the LNZ (15 ± 3.3%, p<0.05) and OSXZ IVC (18 ± 2.1%, p<0.05) (Fig. 2-B). Phosphorylation of IkB demonstrated no difference between the LNZ controls and OSXZ controls. Pressurization increase both the LNZ (111 ± 9.2%, p<0.05) and OSXZ (51 ± 6.6%, p<0.05) levels of phosphorylated IkB, however the pressure induced elevation in the OSXZ IVC was significantly less compared to that seen in the lean animals (Fig. 2-A). The ratio of p-IkB to IkB demonstrated a significant decrease in the basal levels of p-IkB/IkB in the OSXZ IVC (16 ± 5.7%, p<0.05) compared to LNZ controls. Pressurization increase both the LNZ (82 ± 4.7%,p<0.05) and OSXZ (28 ± 5.7%, p<0.05) ratio of p-IkB to IkB. However, the pressure induced elevation in the OSXZ IVC was significantly less compared to that seen in the lean IVC (Fig. 2-C).

### 1.4 Phosphorylation of Fox01 and Fox04

**Fig. 3:**
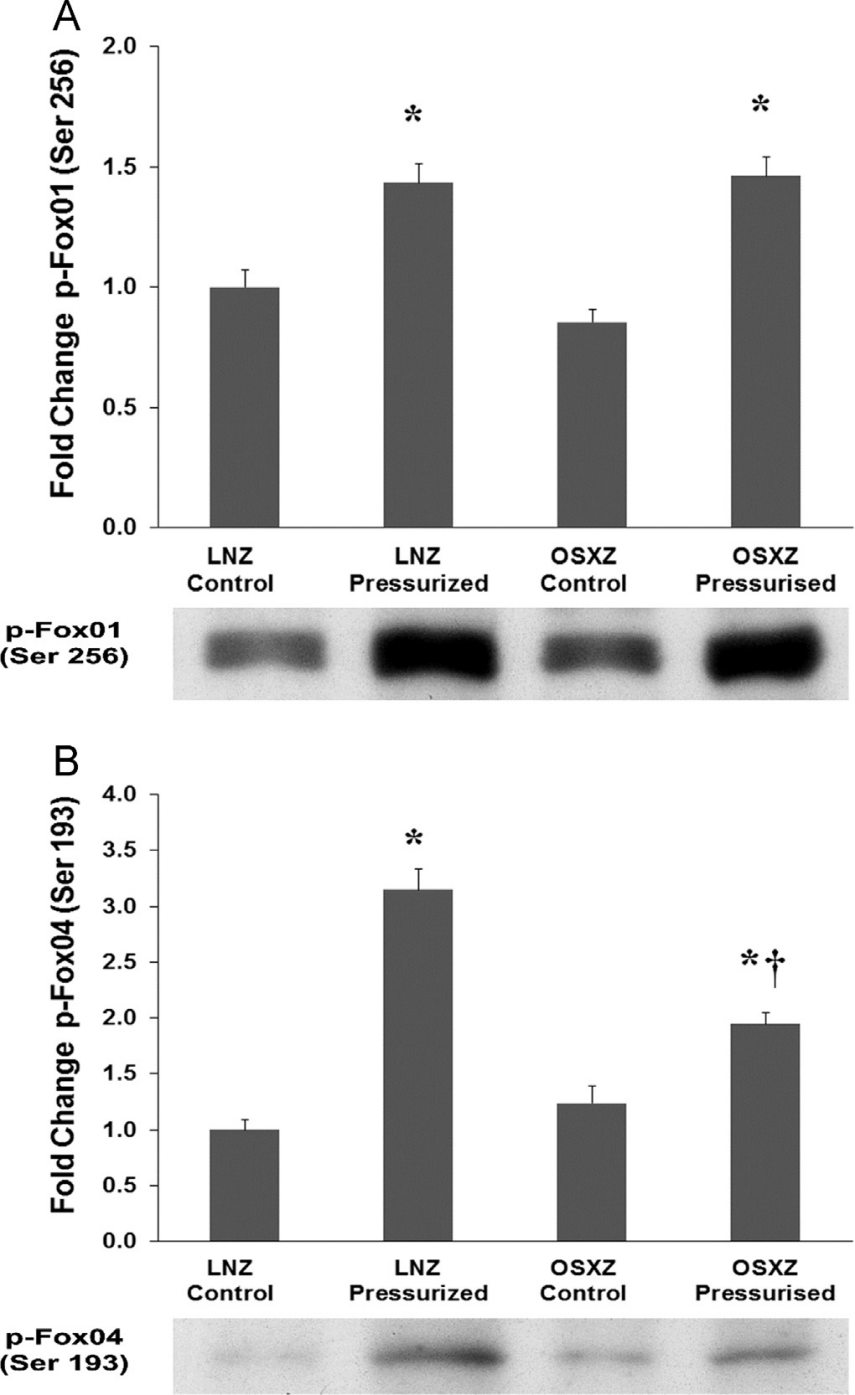
Diabetes alters loading-induced Fox01 and Fox04 phosphorylation in rat inferior vena cava. The basal (control) and pressure-induced phosphorylation of Fox01 (ser 256) and Fox04 (ser 193) in venae cavae from non-diabetic lean Zucker (LNZ) and diabetic obese syndrome X Zucker (OSXZ) rats. * Significantly different from unloaded venae cavae within the same group (p< 0.05). † Significantly different from corresponding LNZ venae cavae (p< 0.05). n = 6/group.

Compared to vessels obtained from the LNZ control animals the phosphorylation of Fox01 [14], [15], [16], [17] basal phosphorylation level was not significantly altered in the OSXZ animals. Pressurization increased Fox01 phosphorylation at the serine 256 residue in the LNZ (43 ± 7.6%, p<0.05) and OSXZ (46 ± 7.7%, p<0.05) IVC (Fig. 3-A). Fox04 basal phosphorylation at the serine 193 residue was not different between the LNZ and the OSXZ control IVCs. Pressurization significantly increased Fox04 phosphorylation in the LNZ (215 ± 18.6%, p<0.05) and OSXZ (95 ± 10.3%, p<0.05). However, the level of OSXZ phosphorylation was significantly less than the magnitude of the LNZ pressure induced phosphorylation (120 ± 28.9%, p<0.05) (Fig. 3-B).

### 1.5 TRAF2

**Fig. 4:**
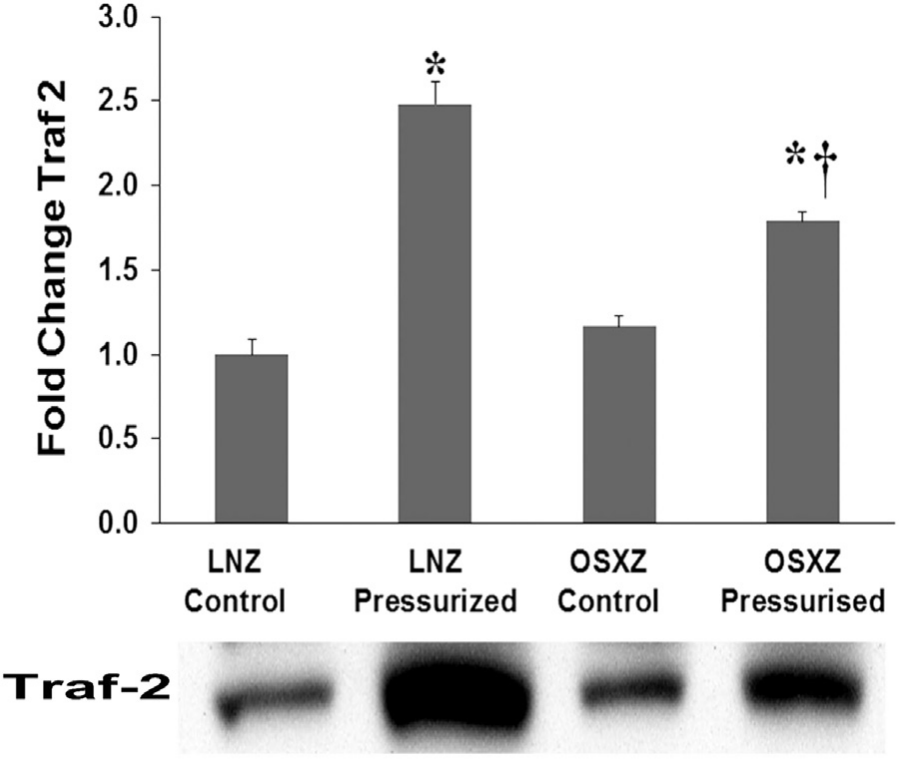
Diabetes alters loading-induced Traf-2 expression in rat inferior vena cava. The basal (control) and pressure-induced expression of Traf-2 in venae cavae from non-diabetic lean Zucker (LNZ) and diabetic obese syndrome X Zucker (OSXZ) rats. * Significantly different from unloaded venae cavae within the same group (p< 0.05). † Significantly different from corresponding LNZ venae cavae (p< 0.05). n = 6/group.

Compared to IVC vessels obtained from LNZ control animals the basal expression of TNF receptor-associated factor 2 (TRAF2) [18] was not significantly alter in the OSXZ animals. Pressurization increased the expression of Traf2 both in the LNZ (148 ± 13.6% p<0.05) and the OSXZ (79 ± 5.6% p<0.05) pressurized IVC. However, the pressure induced increase of Traf2 in the OSXZ was significantly less than that observed in the LNZ (62 ± 5.6% p<0.05) (Fig. 4).

### 1.6 Caspase 12

**Fig. 5:**
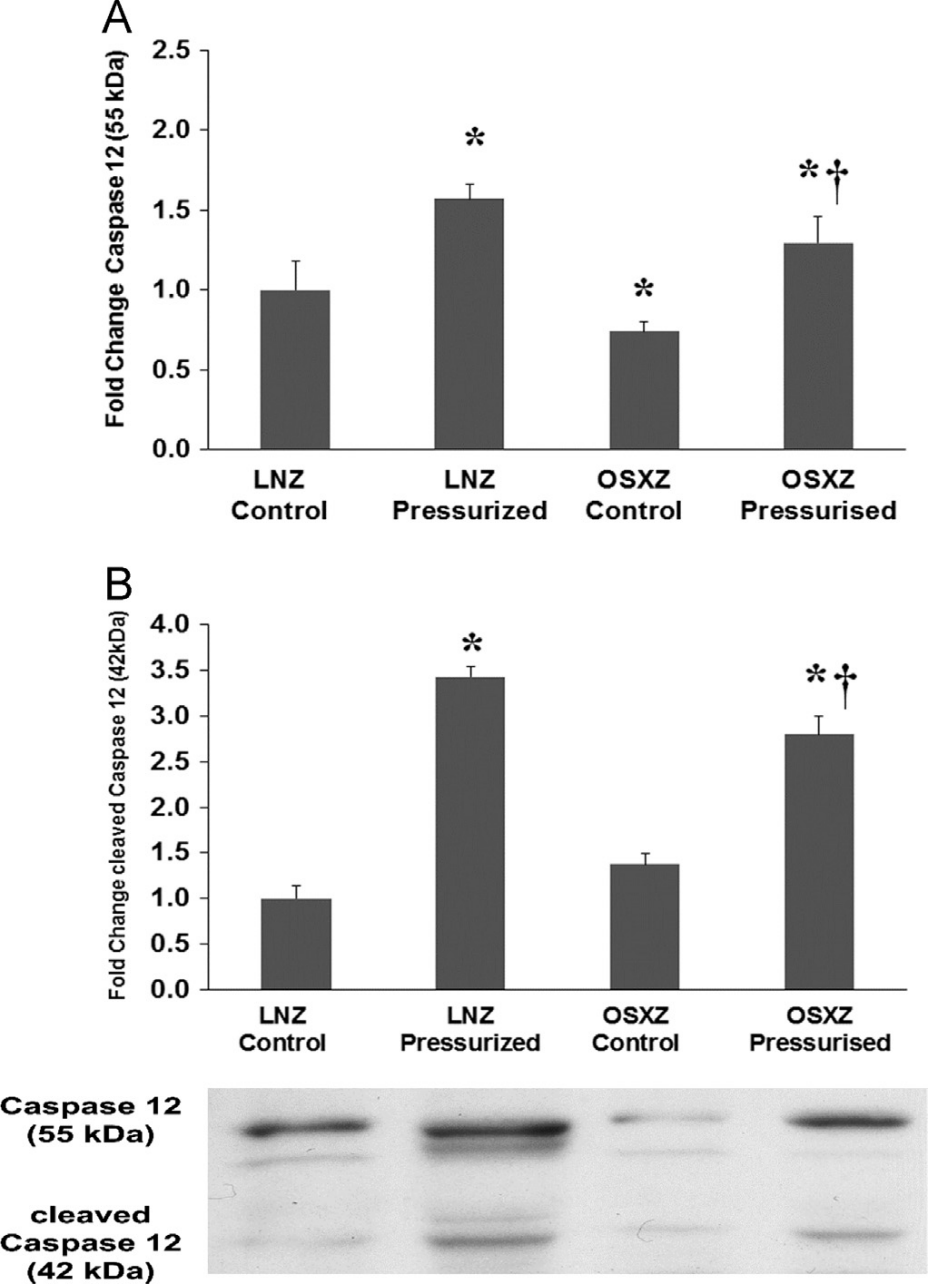
Diabetes alters loading-induced Caspase 12 expression and cleavage in rat inferior vena cava. The basal (control) and pressure-induced expression and cleavage of Caspase 12 in venae cavae from non-diabetic lean Zucker (LNZ) and diabetic obese syndrome X Zucker (OSXZ) rats. * Significantly different from unloaded venae cavae within the same group (p < 0.05). † Significantly different from corresponding LNZ venae cavae (p < 0.05). n = 6/group.

Compared to IVC vessels obtained from the LNZ control animals the basal expression of caspase 12 was significantly reduced in the OSXZ animals (26 ± 6.2%, p<0.05). Expression of caspase 12 was significantly increased in the LNZ pressurized IVC (57 ± 9.2%, p<0.05) and in the OSXZ pressurized IVC (30 ± 16.8%, p*<0.05*). However, the pressure induced increase of caspase 12 observed in the OSXZ was significantly less than the magnitude of the LNZ pressure induced increase (27 ± 16.5% p<0.05) (Fig. 5-A). Compared to IVC obtained from the LNZ control animals, the basal level of cleaved caspase 12 was not significantly altered in the OSXZ animals. Pressurization of the IVC significantly increased caspase 12 cleavage in the LNZ (143 ±10.65%, *p<0.05*) and OSXZ (180 ± 20.7%, p*<0.05*), However, the pressure induced increase of cleaved caspase 12 in the OSXZ was significantly less than that seen in the LNZ (64 ± 30.4%, p<0.05) (Fig. 5-B).

## 2 Experimental design, materials and methods

### 2.1 Animals

All procedures were conducted in strict accordance with the Guide for the Care and Use of Laboratory Animals as approved by the Council of the American Physiological Society and the Animal Use Review Board of Marshall University. Young (10 week, n=12) male lean Zucker (non-diabetic) (LNZ) and young (10 week, n=12) male obese syndrome-X Zucker (diabetic) (OSXZ) rats were obtained from the Charles River Laboratories and barrier housed one per cage in an AAALAC approved vivarium. Housing conditions consisted of a 12 h:12 h dark-light cycle and the temperature was maintained at 22 ±2 °C. Animals were provided food and water *ad libitum*. Rats were allowed to recover from shipment for at least two weeks before the commencement of experimentation during which time the animals were carefully observed and weighed weekly.

### 2.2 Materials

Antibodies against NF-κB p65 [cat #3034], IkBα [cat #9242], p-IKBα (Ser 32/36) (5A5) [cat #9246], p-Fox01 (Ser 256) [cat #9461], p70 S6 Kinase Control Cell Extracts [cat #9203], mouse IgG and rabbit IgG were purchased from Cell Signaling Technology (Beverly, MA). Antibodies against NF-κB p105/p50 (E-10) [sc-8414], and Traf-2 (C-20) [sc-876] were purchased from Santa Cruz Biotechnology (Santa Cruz, CA). Precast 10% and 15% SDS-PAGE gels were purchased from Lonza (Rockland, ME). Enhanced chemiluminescence (ECL) western blotting detection reagent was purchased from Amersham Biosciences (Piscataway, NJ). Restore western blot stripping buffer was obtained from Thermo scientific (Rockford, IL) and 3T3 cell lysates from Santa Cruz Biotechnology (Santa Cruz, CA). All other chemicals were from Sigma (St. Louis, MO).

### 2.3 Inferior vena cava preparation

Rats were anesthetized the rats using a ketamine-xylazine (4:1) cocktail (50 mg/kg ip) and supplemented as necessary for reflexive response. The ventral surface of the thorax was shaved and midline laparotomy was performed. The inferior vena cava was isolated and the *in situ* length measured as the distance from the right atrium to the diaphragm.

After removal, the venae cavae was cleaned of the extra connective tissue and cannulated onto polystyrene tubing (outside diameter 3.0 mm; inner diameter 2.6 mm) with the aid of a dissection microscope. Vessels were securely mounted using 4.0 silk sutures and the vessel length was adjusted after mounting on to the apparatus using micromanipulator to coincide with the *in situ* resting length.

All dissection and mounting procedures were performed rapidly and with care to prevent stretching or tearing of the inferior venae cavae. Vessel manipulation was completed in oxygenated Krebs-Ringer bicarbonate (KRB) buffer maintained at 37 °C. After mounting, venae cavae were allowed to equilibrate in the vessel chamber for at least one hour before pressure loading. Mounted vessels were subjected to 120 mm Hg of pressure for 30 min to examine the effect of increased loading on signal transduction in the inferior vena cavae.

During the incubation, vessels were perfused with oxygenated (95% O_2_, 5% CO_2)_ KRB maintained at 37 °C. Perfusion was accomplished using a peristaltic pump with the flow rate set at 11.1 ml/min resulting in a shear stress of ~0.5 dynes/cm^2^. The intraluminal pressure was controlled by adjusting the air pressure introduced into a fluid reservoir. The system was calibrated prior to experiment. System pressure was monitored using pressure transducers (Gould model P231D) situated before entry and after exit from the mounted vessel. During the loading procedure, the pressure in the vessels was gradually raised in a step-wise fashion (10 mmHg every 1 min) to a mean arterial pressure of 120 mmHg.

### 2.4 Immunoblot analysis

Inferior vena cavae were snap-frozen in liquid nitrogen at the end of each experiment. Protein isolates were prepared from the collected venae cavae by pulverizing the samples under liquid nitrogen using a mortar and pestle and washed three times with ice cold phosphate buffered saline (PBS). T-PER (2 mL/1 g tissue weight) (Pierce, Rockford, IL) supplemented with 100 mM NaF, 1 mM Na_3_VO_4_, 2 mM PMSF 1 μg/ml aprotinin, 1 μg/ml leupeptin, and 1 μg/ml pepsatin was used to extract proteins as detailed by the manufacturer. After centrifugation (1000*g*×10 min), the supernatant was collected and the concentrations of the proteins in the homogenates were determined in triplicate using the Bradford method (Pierce) with bovine serum albumin as a standard. Samples were diluted to a concentration of 1.5 mg/mL in SDS-loading buffer and boiled for 5 minutes. Thirty micrograms of total protein for each sample were separated on 10% or 15% SDS-PAGE gels, transferred onto Hybond nitrocellulose membranes (Amersham Biosciences, Piscataway, NJ) using standard conditions, and stained with Ponceau S to verify transfer and equal loading of lanes.

Membranes were blocked in buffer (5% nonfat dry milk in TBST) for 1 hour at room temperature, washed (TBST, 3×5 min), and incubated in primary antibody overnight at 4° C. Unbound antibody was removed by washing the membranes (TBST, 3×5 min) and the membranes were incubated in horseradish peroxidase (HRP)-linked secondary antibodies for 1 hour at room temperature and rewashed (TBST, 3×5 min). Protein bands were visualized by enhanced chemiluminescence (ECL) Western blotting detection reagent (Amersham Biosciences, Piscataway, NJ) and quantified by densitometry using a flatbed scanner (Epson Perfection 3200 PHOTO) and Imaging software (AlphaEaseFC).

The exposure time was adjusted to keep the integrated optical densities within a linear and non-saturated range. Molecular weight markers (Cell Signaling) were used as mass standards and NIH 3T3 cell lysates were included as positive controls. Membranes were stripped using “Restore” western blot stripping buffer as detailed by the manufacturer. Membranes were interrogated and confirmed for the absence of the residual antibody binding using the ECL reagent after which the membranes were washed (TBST, 3×5 min) before reprobing. Experimental error associated with membrane stripping and reprobing was minimized by randomizing the antibody incubations between experiments.

### 2.5 Data analysis

Data were analyzed using Sigma Stat 3.0 statistical software and the results are presented as mean ± SEM. A one-way analysis of variance (ANOVA) was performed for overall comparisons followed by the Student-Newman-Keuls *post hoc* test to determine differences between groups. The level of significance accepted *a priori* was < 0.05.
