# Supplementary material for: Diabetes alters vascular mechanotransduction data: Pressure-induced regulation of Nf-kapa-B p65 and translational associated signaling in the rat inferior vena cava
Source: Data Brief. 2017 Sep 1;14:676–85. doi: 10.1016/j.dib.2017.08.025 (PMC5596213; doi:10.1016/j.dib.2017.08.025)
Supplement: Supplementary file 1 — Supplementary material [file mmc1.pdf]

AUTHOR DECLARATION

We wish to confirm that there are no known conflicts of interest associated with this publication and there has been no significant financial support for this work that could have influenced its outcome.

We confirm that the manuscript has been read and approved by all named authors and that there are no other persons who satisfied the criteria for authorship but are not listed. We further confirm that the order of authors listed in the manuscript has been approved by all of us.

We confirm that we have given due consideration to the protection of intellectual property associated with this work and that there are no impediments to publication, including the timing of publication, with respect to intellectual property. In so doing we confirm that we have followed the regulations of our institutions concerning intellectual property.

We further confirm that any aspect of the work covered in this manuscript that has involved either experimental animals or human patients has been conducted with the ethical approval of all relevant bodies and that such approvals are acknowledged within the manuscript.

We understand that the Corresponding Author is the sole contact for the Editorial process (including Editorial Manager and direct communications with the office). He/she is responsible for communicating with the other authors about progress, submissions of revisions and final approval of proofs. We confirm that we have provided a current, correct email address which is accessible by the Corresponding Author and which has been configured to accept email from rice9@marshall.edu .

Signed by all authors as follows: [LIST AUTHORS AND DATED SIGNATURES ALONGSIDE]

Kevin M. Rice 7-25-2017

Nandini D.P.K. Manne 7/25/2017

Ravikumar Arvapalli 7/25/2017

Gautam K. Ginjupalli 7/25/2017

Eric R. Blough 7/25/17
